# Roles of non-coding RNAs in the hormonal and nutritional regulation in nodulation and nitrogen fixation

**DOI:** 10.3389/fpls.2022.997037

**Published:** 2022-10-18

**Authors:** Kejing Fan, Ching-Ching Sze, Man-Wah Li, Hon-Ming Lam

**Affiliations:** School of Life Sciences and Centre for Soybean Research of the State Key Laboratory of Agrobiotechnology, The Chinese University of Hong Kong, Hong Kong, Hong Kong SAR, China

**Keywords:** nitrogen fixation, nodulation, non-coding RNA, legume, rhizobium, autoregulation of nodulation, nutrient homeostasis, phytohormone

## Abstract

Symbiotic nitrogen fixation is an important component in the nitrogen cycle and is a potential solution for sustainable agriculture. It is the result of the interactions between the plant host, mostly restricted to legume species, and the rhizobial symbiont. From the first encounter between the host and the symbiont to eventual successful nitrogen fixation, there are delicate processes involved, such as nodule organogenesis, rhizobial infection thread progression, differentiation of the bacteroid, deregulation of the host defense systems, and reallocation of resources. All these processes are tightly regulated at different levels. Recent evidence revealed that non-coding RNAs (ncRNAs), including microRNAs (miRNAs), long non-coding RNAs (lncRNAs), and circular RNAs (circRNAs), participate in these processes by controlling the transcription and translation of effector genes. In general, ncRNAs are functional transcripts without translation potential and are important gene regulators. MiRNAs, negative gene regulators, bind to the target mRNAs and repress protein production by causing the cleavage of mRNA and translational silencing. LncRNAs affect the formation of chromosomal loops, DNA methylation, histone modification, and alternative splicing to modulate gene expression. Both lncRNAs and circRNAs could serve as target mimics of miRNA to inhibit miRNA functions. In this review, we summarized and discussed the current understanding of the roles of ncRNAs in legume nodulation and nitrogen fixation in the root nodule, mainly focusing on their regulation of hormone signal transduction, the autoregulation of nodulation (AON) pathway and nutrient homeostasis in nodules. Unraveling the mediation of legume nodulation by ncRNAs will give us new insights into designing higher-performance leguminous crops for sustainable agriculture.

## Introduction

Legumes are important crops that benefit the world in diverse ways. Chief among them is the ability to form a specific organ called the root nodule to convert atmospheric nitrogen into bioavailable nutrients. Nodulation and nitrogen fixation are complicated processes that are regulated at different levels to maximize the benefits to the host and the microsymbiont. The interaction between the legume host and rhizobia is normally initiated with the release of flavonoids from the plant root to attract the rhizobium (Cohn et al., 1998), which is then entrapped in the root hair cells. The rhizobium multiplies to form infection foci, from which infection threads are developed toward the cortical cells to facilitate rhizobial entry (Oldroyd and Downie, 2008). Alternatively, the rhizobium can invade the cortical cells through cracks on the epidermis (Oldroyd and Downie, 2008), and produce nodulation factors (Nod factors [NFs]), which are recognized by the host to start nodule formation (Cohn et al., 1998). Nod factor perception and transduction at the epidermis trigger root cell swelling, Ca^2+^ spiking (Ehrhardt et al., 1996) and the activation of early nodulation genes to advance the early events of nodulation (Oldroyd and Downie, 2008). Several kinases, transcription factors (Oldroyd and Downie, 2008) and other genes (Stacey et al., 2006) are involved in the Nod factor signaling pathway. Upon sensing epidermal responses, the cortical cells divide and form the nodule primordium where nodule organogenesis and development take place. The rhizobium then enters the cortical cells and is encapsulated within the plant cell membrane. The cell membrane and rhizobium differentiate together to form a symbiosome (Oldroyd and Downie, 2008). A nodule then develops, within which the rhizobium performs nitrogen fixation (Oldroyd and Downie, 2008). Several phytohormones are essential for maintaining highly effective nitrogen fixation (Oldroyd and Downie, 2008).

Determinate and indeterminate nodules are developed in different species of legumes. For instance, soybean forms determinate nodules and alfalfa forms indeterminate ones (Mao et al., 2013). Determinate nodules originate from the middle or outer cortex of the plant with a transient nodule meristem that is absent in distant zonation while indeterminate nodules develop from the inner cortex or pericycle with a persistent nodule meristem that is spatially separated into five zones (Kohlen et al., 2018). These two kinds of nodules are suggested to respond differently to auxin or other diffusible signals during nodule development (Kohlen et al., 2018).

Biological nitrogen fixation (BNF) is crucial for maintaining nitrogen cycling in the biosphere. It converts atmospheric nitrogen into ammonia (Long, 1989), an ultimate goal of legume-rhizobium symbiosis. This symbiosis fixes 21 metric tons of nitrogen annually (Herridge et al., 2008) and increases bioavailable nitrogen, bringing about agricultural, natural and socio-economic benefits. Considering the high energy cost of producing synthetic fertilizers, the substitution of synthetic fertilizer with biological nitrogen fixation has become a crucial element of sustainable agriculture (Foyer et al., 2016).

Non-coding RNAs (ncRNAs) are functional transcripts without translation potential (Mattick and Makunin, 2006). Major groups of regulatory ncRNAs in plants are small RNAs (20-24 nt), which include microRNAs (miRNAs) and small interfering RNAs (siRNAs) (Borges and Martienssen, 2015), long non-coding RNAs (lncRNAs) (>200 nt) (Wang T. et al., 2017), and circular RNAs (circRNAs) (Ye et al., 2015).

MiRNAs are transcribed by RNA polymerase II (Pol II) in the nucleus to produce single-stranded primary miRNAs, which are then processed by DICER-LIKE (DCL) 1 protein to form the mature miRNA duplex. MiRNAs form a silencing effector complex with specific ARGONAUTE (AGO) proteins (Borges and Martienssen, 2015). The complementary sequences of miRNAs to their target mRNAs guide the complex to perform post-transcriptional gene-silencing by target mRNA cleavage or translational repression (Borges and Martienssen, 2015). For siRNAs, an endogenous gene is transcribed by Pol II or Pol IV and might be processed by RNA-DEPENDENT RNA POLYMERASEs (RDRs). The transcripts are then cleaved by DCLs to form siRNAs or secondary siRNAs (Borges and Martienssen, 2015). MiRNA-mediated gene-silencing might also produce secondary siRNAs (Manavella et al., 2012). SiRNAs are categorized into hairpin-derived siRNAs (hp-siRNAs), natural antisense *trans*- or *cis*-acting siRNAs (natsiRNAs), heterochromatic siRNAs (hetsiRNAs) and secondary siRNAs consisting of *trans*-acting siRNAs (ta-siRNA), phased siRNAs (phasiRNAs) and epigenetically activated siRNAs (easiRNA) (Borges and Martienssen, 2015). SiRNAs can silence their target genes by mRNA cleavage, transcriptional (Baulcombe, 2004) and translational repression, and DNA methylation (Borges and Martienssen, 2015).

Plant lncRNAs are classified into long intergenic ncRNAs (lincRNAs), intronic ncRNAs (incRNAs) and antisense RNAs and natural antisense transcripts (NATs) (Wang J. et al., 2017). LncRNAs are produced by Pol I, II, III and two plant-specific enzymes, Pol IV and V (Wang J. et al., 2017). LncRNAs can act *in trans* and *in cis* and they function by affecting the formation of chromosomal loops, DNA methylation, histone modification, and alternative splicing to modulate promoter and terminator activities and regulate gene expressions (Statello et al., 2020). LncRNAs can also act as miRNA sponges which served as the mimicry of the miRNA target to sponge up the miRNA, which help to control miRNA activities in gene regulation (Zhu and Wang, 2012).

CircRNAs are single-stranded RNAs with a phosphodiester linkage at the 3′ and 5′ ends that forms a stable circular molecule (Chen, 2016). CircRNAs are mainly produced by back-splicing and can be generated from exons (exonic circRNA), introns (intronic circRNA), intergenic regions (Ye et al., 2015) and transcripts of transposons (Chen et al., 2018). It can regulate other linear transcripts or genes by acting as target mimics of miRNAs to inhibit miRNA functions (Chen, 2016), modulating gene expression post-transcriptionally, or mediating alternative splicing (Chen, 2016).

Various ncRNAs are reported to play roles in plant development, plant immunity and responses to abiotic and biotic stress (Wang T. et al., 2017; Chand Jha et al., 2021), indicating the importance of ncRNAs in plants. There are also many studies illustrating that ncRNAs are critical regulators in the nodulation process.

Several legume species, such as soybean and peanut, are important crops worldwide as they are the major sources of proteins and carbohydrates. The symbiosis between legumes and rhizobia ensures the bioavailability of nitrogen, which is an essential part of the nitrogen cycle and contributes to sustainable agriculture and ecological balance (Salvagiotti et al., 2008). Up to now, many nodulation-related regulators have been identified. Among them, ncRNAs have been proven to play key roles in this process. In this review, we described the roles of miRNAs, lncRNAs and circRNAs in controlling nodulation through the regulation of hormone signal transduction, the AON pathway and nutrient transportation. With the widespread application of next-generation sequencing, more and more ncRNAs are identified to be involved in the interactions between legumes and rhizobia. MiRNAs play critical roles in nodule formation, nodule development and nitrogen fixation in mature nodules, while the functions of lncRNAs and circRNAs are still not well defined.

## Identification of non-coding RNAs governing nodulation and nitrogen fixation in legumes by high-throughput sequencing

With the advances in sequencing methods, more and more research has been carried out to identify ncRNAs governing nodulation and nitrogen fixation. Up to now, miRNAs, siRNAs, lncRNAs and circRNAs have been identified in a number of legume species, especially in soybean, *Medicago truncatula*, common bean (*Phaseolus vulgaris*) and *Lotus japonicus* (Wang et al., 2020; Chand Jha et al., 2021).

### MiRNAs

MiRNA is the most studied class of ncRNAs, and is the most conserved type of ncRNAs among plant species (Li and Mao, 2007; De Lima et al., 2012). Most plant species usually have both conserved and species-specific miRNAs (De La Rosa et al., 2020). Even within the same legume species, different germplasms can harbor their own specific miRNAs. For example, cultivated soybean (*Glycine max*) and wild soybean (*Glycine soja*) have their own specific miRNAs (Zeng et al., 2012; Sun Y. et al., 2016). The functions of conserved miRNAs among plant species are quite similar (Paul et al., 2015; Li and Zhang, 2016), mostly involved in controlling development and stress responses (Rubio-Somoza and Weigel, 2013; Teotia and Tang, 2015; Fan et al., 2021). The expression profiles of conserved miRNAs vary a lot across plant species and organs (Tsikou et al., 2018). Since biological nitrogen fixation is a unique feature in legumes, (Salvagiotti et al., 2008), research on miRNAs in legumes is often focused on this symbiotic function. For example, 382 miRNA candidates, including 154 known mature miRNAs, 57 variants of known miRNAs and 171 novel miRNAs, were identified in soybean roots and nodules (Fan et al., 2021). About 68.1% of the miRNAs were differentially expressed in mature nodules (Fan et al., 2021) inferring their potential roles in nitrogen fixation and nodule maintenance. In *M. truncatula*, 73 miRNA belonging to 27 known miRNA families and 100 new miRNA candidates were identified (Lelandais-Brière et al., 2009). These miRNAs also showed differential expression profiles in roots and nodules (Lelandais-Brière et al., 2009). In chickpea nodules, 91 miRNAs were identified, including 84 conserved and 7 novel miRNAs (Tiwari et al., 2021). Among them, 28 miRNAs were legume-specific, of which 13 were chickpea-specific (Tiwari et al., 2021). These studies showed that the miRNA regulation on legume nodules is common among legume species, with diversities among the specific miRNAs involved.

### LncRNAs

Unlike miRNAs, lncRNAs are less conserved among plants (Deng et al., 2018). Compared to animals, plant lncRNAs are generally shorter, and have fewer exons than protein-coding genes (Deng et al., 2018). The abundance of lncRNAs is usually correlated with the number of mRNAs in plant species (Deng et al., 2018). Across species, lncRNAs exhibit a high divergence with conserved genomic positions, but distinct nucleotide sequences (Deng et al., 2018). However, those lncRNAs which are the precursors or targets of miRNAs normally show a certain percentage of sequence conservation among different plant species (Deng et al., 2018). Meanwhile, sequences of lncRNAs within individual species also show some degree of conservation (Deng et al., 2018). For instance, 48,752 and 35, 675 lncRNA gene loci were identified in cultivated soybeans and wild soybeans, respectively (Lin et al., 2020), with ~12,000 loci shared between cultivated and wild soybeans (Lin et al., 2020). However, most of the soybean lncRNAs cannot be aligned well with the genomes of other legume species, showing that lncRNAs are more diverged across species than other types of ncRNAs (Lin et al., 2020).

### CircRNAs

The biogenesis of circRNAs is conserved in both plants and animals, which depends on RNA polymerase II-mediated transcription and back-splicing reactions of the mRNA precursor (Sun X. et al., 2016; Zhao et al., 2019a). However, due to alternative circularization, circRNAs are rich in isoforms (Zhang et al., 2014; Zhao et al., 2019a). In soybean, 5,372 circRNAs were identified by high-throughput sequencing, with 80% of them being paralogous circRNAs formed from paralogous genes (Zhao et al., 2017). CircRNAs showed tissue-specific expression patterns and were potentially involved in many biological processes, such as multi-organism processes and developmental processes (Zhao et al., 2017). In common bean, 8,842 high-confidence circRNAs were identified, with 3,448 of them specifically expressed during symbiosis, peaking at the nitrogen-fixing stage (Wu et al., 2020). These findings provide evidence that circRNAs participate in legume nodulation.

## NcRNAs regulate nodulation through mediating phytohormone signaling pathways

Phytohormones play important roles in plant growth and development. Most of them were shown to be involved in nodulation and nitrogen fixation, especially auxins, cytokinins (CK), gibberellins (GA), ethylene (ET) and jasmonates (JA). Recently, miRNAs and siRNAs, both small ncRNAs, have been identified to control nodule formation and development by mediating phytohormone signal transduction. The specific ncRNAs involved in hormone signal transduction and the references are listed in **Table 1**.

**Table 1:** Non-coding RNAs (ncRNAs) related to hormone signal transduction.

Type of ncRNA	Name	Related hormonal pathways	Targets	Species	References
miRNA	miR156	Gibberellic acid, cytokinin, auxin, ethylene	SPL	*Medicago sativa*	(Feyissa et al., 2021)
	miR160	Auxin	ARF10/ARF16/ARF17 repressor	*Glycine max, Medicago truncatula*	(Bustos-Sanmamed et al., 2013; Nizampatnam et al., 2015)
	miR164	Auxin	NAC1	*Medicago truncatula*	(D’haeseleer et al., 2011)
	miR166	Gibberellic acid	GAI	*Medicago sativa*	(Ma J. et al., 2020)
	miR167	Auxin	ARF8	*Glycine max*	(Wang et al., 2015)
			ARF6, ARF8	*Medicago sativa*	(Ma J. et al., 2020)
	miR171b	Ethylene	ERF genes	*Cicer arietinum L.*	(Garg et al., 2019)
	miR172	Ethylene	ERF	*Medicago sativa*	(Ma J. et al., 2020)
	miR319d	Jasmonates	TCP10	*Phaseolus vulgaris*	(Martín-Rodríguez et al., 2018)
	miR390	Auxin	TAS3	*Medicago truncatula*	(Hobecker et al., 2017)
	miR393	Auxin	GmTIR1- and GmAFB3	*Glycine max*	(Mao et al., 2013; Cai et al., 2017)
	miR394	Cytokinin	*Histidine phosphotransferase (HP)*	*Cicer aritienum*	(Tiwari et al., 2021)
	miR397	Abscisic acid	PYL8	*Medicago sativa*	(Ma J. et al., 2020)
	miR5037	Gibberellic acid	GRAS family transcription factors	*Medicago sativa*	(Li et al., 2018; Zhang et al., 2021a)
	ahy_novel_miRn23	Abscisic acid	CYP707A1 and CYP707A3	*Arachis hypogaea*	(Chen et al., 2019)
	ahy_novel_miRn112	Brassinosteroids	BR1	*Arachis hypogaea*	
	ahy_novel_miRn38	Jasmonates	TCP4	*Arachis hypogaea*	
	miR1514	Auxin	NAM transcripts	*Glycine soja*	(Zeng et al., 2012)
	ahy_novel_miRn25	gibberellic acid	GAMYB	*Arachis hypogaea*	(Chen et al., 2019)
	ahy_novel_miRn112	Auxin	NAM/CUC, NAC, NAD and CUC2	*Arachis hypogaea*	
	novel-m1245-3p	Ethylene	Predicted target: Glyma.13G123100.1	*Glycine max*	(Liu et al., 2020)
siRNA	TAS3(Ta-siRNA)	Auxin	ARF3a, ARF3b and ARF4 transcript	*Lotus japonicus*	(Li et al., 2014)
	TasiARFs(Ta-siRNA)	Auxin	ARF2, ARF3, ARF4a and ARF4b	*Medicago truncatula*	(Hobecker et al., 2017; Kirolinko et al., 2021)
lncRNA	MSTRG.24254.1	Auxin	LOC112696267	*Arachis hypogaea*	(Zhao et al., 2019b)
	XR_001589909.1	Auxin	Predicted transcript: XM_016098404.1	*Arachis hypogaea L.*	(Ma X. et al., 2020)
	XR_001621694.1	Gibberellic acid	Predicted transcript: XM_016349993.1	*Arachis hypogaea L.*	
	MSTRG.2711.1	Auxin	gma-miR5369	*Glycine max*	(Khoei et al., 2021)
	MSTRG.26588.1	Jasmonates	gma-miR9725	*Glycine max*	
	MSTRG.19778.1	gibberellic acid	Glyma.13G069900.1	*Glycine max*	
	scaffold876:319353-320260	Auxin	car-miRNA015	*Cicer arietinum L.*	(Kumar et al., 2021)
	Ca7:1605751-1607467	gibberellic acid	Cat-miR159g-3p	*Cicer arietinum L.*	
	Ca8:5379085-5379448	Ethylene	Cat-miR172c.2	*Cicer arietinum L.*	
	TCONS_00030280 and TCONS_00068008	Ethylene	miR169 l-3p	*Glycine max*	(Zhang et al., 2020; Zhang et al., 2021b)
circRNA	Gm03circRNA1785	Auxin	gma-miR167c	*Glycine max*	(Zhao et al., 2017)
	gma_circ_0000352	Ethylene	gma-miR5678	*Glycine max*	

### Auxin

Auxin signaling plays crucial roles in nodule formation and development (Kohlen et al., 2018). The alteration in auxin homeostasis upon rhizobial infection was shown to be a prerequisite for nodule formation (Mathesius and Mathesius, 2008). Specifically, the inhibition of polar auxin transport and down-regulation of the auxin-inducible promoter, GH3, are associated with the formation of the nodule primordium, where low levels of auxin are required for the initiation of nodule formation (Mathesius et al., 1998; Pacios-Bras et al., 2003; De Billy et al., 2001; Grunewald et al., 2009). During nodule development, auxin is responsible for controlling the cell cycle and nodule maturation (Kondorosi et al., 2005; Nizampatnam et al., 2015).

MiRNAs are important regulators in the auxin signaling pathway. MiRNAs, such as miR160, miR164, miR167, miR390 and miR393, were reported to control nodulation by affecting auxin signaling (Nizampatnam et al., 2015; Wang et al., 2015; Yan et al., 2015; Hobecker et al., 2017). MiR160, miR167 and miR390 target different auxin response factors (ARF) and perform different regulatory roles in nodulation (Nizampatnam et al., 2015; Wang et al., 2015; Hobecker et al., 2017). Auxin directly binds to TRANSPORT INHIBITOR RESPONSE1 (TIR1)-like F-box proteins to form part of an Skp, Cullin, F-box (SCF)-type ubiquitin ligase complex that regulates the degradation of auxin/indole-3-acetic acid (Aux/IAA) (Dharmasiri et al., 2005; Kepinski and Leyser, 2005; Turner et al., 2013). Aux/IAAs are repressor proteins that form complexes with ARFs to repress the expression of auxin-responsive genes (Liscum and Reed, 2002; Remington et al., 2004). The degradation of Aux/IAAs caused by TIR1 in an auxin dose-dependent manner leads to the de-repression/expression of auxin-responsive genes (Gray et al., 2001; Schwechheimer et al., 2001). Overexpression of miR160 could inhibit the expressions of the auxin response repressing transcription factors, *ARF10*/*16*/*17*, to make the root hypersensitive to auxin and reduce sensitivity to cytokinin (Turner et al., 2013; Nizampatnam et al., 2015). Auxin hypersensitivity leads to a reduction in nodule primordium formation (Turner et al., 2013). Therefore, overexpression of miR160 reduces the nodule numbers, which can be restored by exogenous cytokinin application (Nizampatnam et al., 2015). Knockdown of miR160 results in reduced sensitivity to auxin and enhanced sensitivity to cytokinin to increase nodule formation and delay nodule maturation (Nizampatnam et al., 2015). Exogenous auxin rescues the proper nodule formation and maturation in miR160 knockdown roots (Nizampatnam et al., 2015). At the early stage of nodule formation in soybean, a low level of miR160 promotes nodulation, and high miR160 levels in nodule development could stimulate nodule maturation (Nizampatnam et al., 2015). In contrast to miR160, miR167c is induced by rhizobium inoculation and positively mediates nodulation efficiency under low microsymbiont density by targeting the negative regulators of nodulation, *ARF8a* and *ARF8b*, that impair nodule formation (Wang et al., 2015). The miR167c overexpression reduces the root sensitivity to auxin, while the knockdown of miR167c enhances the root sensitivity to auxin (Wang et al., 2015). MiR167c also positively controls the expression of a set of nodulation marker genes, like *ENOD40* (Wang et al., 2015).

MiR390, an evolutionarily conserved miRNA, targets the *Trans-Acting Short Interference RNA3* (*TAS3*) and causes the cleavage of *TAS3* by ARGONAUTE7 at the 3’ ends most proximal site to produce ta-siRNA (Hobecker et al., 2017). *TAS3*-derived ta-siRNAs directly control the cleavage of complementary mRNAs encoding *ARF2/3/4* (Hobecker et al., 2017; Kirolinko et al., 2021). Knockdown of *ARF2/3/4* reduces the initiation and progression of infection events to inhibit nodule formation and downregulate the Nodulation Signaling Pathway 2 (*NSP2*) (Kirolinko et al., 2021). At the early stage of nodulation, the expression level of miR390 and tasiRNAs are at a low level, whereas *TAS3* and *ARF2/3/4* are substantially highly expressed to promote the nodule formation (Hobecker et al., 2017).

Other than regulating *ARFs*, nodulation can also be regulated by direct targeting of auxin-responsive genes by miRNAs to silence their expressions (Guo et al., 2005; D’haeseleer et al., 2011; Yan et al., 2015). In *Medicago truncatula*, miR164 causes a reduction in nodule numbers by causing the cleavage of the mRNA of the auxin-induced transcription factor, *NAC1* (Guo et al., 2005; D’haeseleer et al., 2011). MiR393 also works as a negative regulator in nodule formation by directly targeting the transcripts of the auxin receptor, TIR1/AFB, to alter auxin signaling in roots (Cai et al., 2017; Kohlen et al., 2018). Overexpression of *GmTIR1A* and *GmTIR1C*, target genes of miR393, could increase the nodule numbers through modulation of cellular response to auxin (Cai et al., 2017).

### Cytokinin

CK plays a crucial role in the initiation of nodule organogenesis (Gamas et al., 2017). Upon rhizobial infection, nodulation factor (NF) induces the accumulation of CK (Van Zeijl et al., 2015). Exogenous application of a low concentration of CK to soybean could enhance nodule formation, while the application of a high concentration of CK did the opposite (Mens et al., 2018). Interestingly, CK plays an important role in the AON pathway (Sasaki et al., 2014). In *Lotus japonicus*, the CLE-RS1/2-HAR1 signaling pathway stimulates the transportation of shoot-derived CK to the root to suppress nodulation (Sasaki et al., 2014). CLE production and the whole AON pathway are regulated by miR156b and miR172c (Wang et al., 2014; Wang et al., 2019; Yun et al., 2022). The high expression of miR172c activates the production of *ENOD40* and *GmCLE* through inhibiting its target gene, *Nodule Number Control 1* (*GmNNC1*) (Wang et al., 2014). *GmNNC1* is a transcription repressor of *ENOD40* and *GmCLE* (Wang et al., 2014). Therefore, the shoot-derived CK regulation on nodulation is also controlled by miR156b and miR172c (Yun et al., 2022). The detailed information about ncRNAs regulation on the AON pathway will be introduced in section 4.

### Gibberellin

Exogenous application of GA demonstrated both positive and negative roles of GA in legume nodulation (Hayashi et al., 2014). Recently, *gibberellin 20-oxidase 1a* (*GA20ox1a*), *GA3ox1a* and *GA2ox1a* were shown to be induced by rhizobial infection in soybean roots and the induction of these three genes was required for nodule organogenesis (Chu et al., 2022). A reduced level of GA led to aberrant rhizobial infection and inhibited nodule formation (Chu et al., 2022). In soybean, miR166 targets a transcription factor *ATHB14-LIKE*, which directly represses the transcription of the GA biosynthesis genes *GmGA1* and *GmGA2*, and activates the transcription of catabolic gene *GIBBERLLIN 2 OXIDASE 2* (*GmGA2ox2*) (Zhao et al., 2022). Therefore, miR166 could regulate the level of bioactive gibberellic acid (GA3) (Zhao et al., 2022). In Medicago, miR166a targets several class-III homeodomain-leucine zipper (*HD-ZIP III*) genes: *MtHB8*, *MtCNA1* and *MtCNA2* (Boualem et al., 2008). These three *HD-ZIP III* genes show the strong expressions in nodule primordia followed by in the apical region and vascular bundles in the mature nodules, which suggests they are involved in the new meristem formation and vascular-bundle differentiation (Boualem et al., 2008). Overexpression of miR166a reduces the symbiotic nodule numbers and lateral roots and improves ectopic development of vascular bundles (Boualem et al., 2008). In alfalfa (*Medicago sativa*), transcriptomic analyses found that the overexpression of miR156 improved the expressions of GA receptors and the GA-regulated family of proteins, promoting nitrogen fixation activity (Aung et al., 2017). In peanut (*Arachis hypogaea* L.), an lncRNA, XR_001621694.1, was identified to target a GA receptor gene, *GID1C-like*, to regulate the GA signaling pathway, which may in turn regulate nodulation (Ma X. et al., 2020).

### Ethylene

ET plays an important role in the nodulation process as a negative regulator (Guinel, 2015). Rhizobium-derived flagellin-like molecules (flg22) could trigger the production of ET (Nakagawa et al., 2011), activating the expression of ethylene response factor (ERF)-encoding genes, which in turn upregulate the expression of Pathogenesis-Related (PR) proteins to inhibit the progress of the infection thread (Breakspear et al., 2015; Guinel, 2015).

In chickpea, miR171b expression was negatively correlated with that of *ERF* (*Ca_12975*), and was shown to regulate nodule formation (De Luis et al., 2012; Garg et al., 2019). Another miRNA, miR5678 from soybean, was predicted to target an ethylene receptor 1-related gene (*Glyma.12G241700*) and its sequence is reverse-complementary to a circRNA, Gma-circ_0000352 (Wang et al., 2020), suggesting that Gma-circ_0000352 may affect nodulation through the interaction with an miRNA.

### Jasmonate

JAs serve either positive or negative roles in nodulation in a species-specific manner, by affecting the formation of infection threads and the expression of nodulation-related genes, such as *ENOD11* (Sun et al., 2006; Ferguson and Mathesius, 2014). Studies on the relationship between the JA signaling pathway and ncRNAs are limited. One example is found in common bean, where miR319d indirectly suppresses the expression of a JA biosynthetic gene, *lipoxygenase 2* (*LOX2*) through the direct inhibition of *TEOSINTE BRANCHED/CYCLOIDEA/PCF10* (*TCP10*) (Martín-Rodríguez et al., 2018), thus exhibiting a role in regulating the JA content during nodulation. As a consequence, an increase in miR319d expression contributed to rhizobial infection but reduced nodule formation (Martín-Rodríguez et al., 2018). MiR319d expression level is induced after rhizobium infection in roots to promote nodule formation (Martín-Rodríguez et al., 2018).

### Coordinated regulation of ncRNAs in hormones, biotic and abiotic stresses

Plant hormones could mediate stress responses when the plant encounter stresses like salinity, drought, heat, cold and heavy metals, and bacterial and viral infection. In recent studies, hormone-responsive miRNAs in symbiotic soybeans were also found to be important regulators for abiotic and biotic stresses in plants, which may contribute to the maintenance of nitrogen fixation and nodule formation under stresses. Auxin contributes to plant tolerance to salt, drought, heat stress, and microbe infection (Šečić et al., 2021; Waadt et al., 2022). As the main regulator of auxin signaling, miR160, miR167, miR390 and miR393 were differentially expressed under salt and drought stresses (Kumar, 2014; Jatan and Lata, 2019). MiR160 and miR167 can improve the level of auxin by targeting TIR1 or *ARF* to reset the development program, which in turn counters biomass damage due to salt stress (Yin et al., 2017). Thus, the alternation of the expression of these miRNAs under abiotic stress may affect the nodule functions. In chickpea, miR319 was upregulated by salt stress, while it was also the most down-regulated miRNA in soybean functional nodules upon salt stress (Dong et al., 2013). As mentioned above, a high level of miR319 could reduce the nodule number. Therefore, the downregulation of miR319 under salt stress may protect the nodule formation. Except for abiotic stress, miR160, miR167, miR390 and miR393 are also responsive to the pathogenic bacterium *Pseudomonas syringae* and the pathogenic fungus *Verticillium dahlia* (Šečić et al., 2021). Overexpression of miR160 leads to altered callose deposition and bacteria resistance (Li et al., 2010). MiR393 was induced by flag22 treatment to repress the auxin signaling to restrict *P. syringae* growth (Navarro et al., 2006). In soybean, miR393 was induced by heat-inactivated *Phytophthora sojae* hyphae, and the knockdown of miR393 reduces the defense against *P. sojae* and the production of isoflavonoids, one major group of soybean antimicrobial metabolites (Wong et al., 2014). MiR160, miR167 and miR393 are induced by soybean mosaic virus (SMV), and miR167 could reduce the harm of SMV by targeting *ARF* (Yin et al., 2013). Therefore, miRNAs regulation in both hormones and stresses is required for legume nodule formation and development.

## Regulation of the AON pathway by ncRNAs

Symbiotic nitrogen fixation in legume nodules is a highly energy-intensive process requiring a great amount of carbon in the form of sugars (Ferguson et al., 2019). Therefore, legumes have tight control over nodule numbers to balance the symbiotic relationship with their own development (Ferguson et al., 2019), by maintaining the optimal number of nodules through the AON pathway (Kosslak and Bohlool, 1984; Suzuki et al., 2008; Reid et al., 2011). MiRNAs and lncRNAs are the core regulators in this pathway (Ferguson et al., 2019).

During rhizobial infection, NFs are perceived by NF receptors (NFRs), such as NFR1a/5a, which then activate the expressions of downstream genes, such as *nodule inception* (*NIN*), miR172c and *nodulation signaling pathway* (*NSP*) (Madsen et al., 2003; Madsen et al., 2011). In soybean, besides being regulated by NFR1a/5a, miR172c is also under the control of the transcription factor, *NINa* and miR156b (Wang et al., 2019). *NINa* directly binds to the promoter of miR172c to stimulate its transcription (Wang et al., 2019), whereas miR172c represses its target gene, *Nodule Number Control 1* (*NNC1*) (Wang et al., 2014). *NNC1* encodes a protein that targets the promoter of the early nodulin gene, *ENOD40* (Wang et al., 2014). The *ENOD40* RNA is a special class of RNAs in plants, known as bifunctional RNAs, with both coding and non-coding functions (Röhrig et al., 2002; Ganguly et al., 2021). *ENOD40* initiates the proliferation of root cortical cells and increases nodule numbers (Crespi et al., 1994; Charon et al., 1997; Campalans et al., 2004). In *Pisum sativum*, *ENOD40* shows a high expression level in the nodule primordium and prefixing zones in mature nodules, suggesting that *ENOD40* is involved in nodule development (Matvienko et al., 1994). Furthermore, *ENOD40* encodes peptides that were found to bind to sucrose synthase and could potentially control the enzymatic activity to mediate sucrose use in soybean nodules (Röhrig et al., 2002).

MiR156b is a negative regulator of nodulation by targeting *SQUAMOSA promoter-binding protein-like 9D* (S*PL9d*). *SPL9d* improves nodulation through binding to the promoter of miR172c and stimulating its expression. *SPL9d* is co-expressed with nodulation marker genes, *NINa* and *ENOD40-1*, during nodule formation and development (Yun et al., 2022). Therefore, the miR156b-SPL9d module and *NINa* both act as upstream master regulators of soybean nodulation.

**Figure 1:**
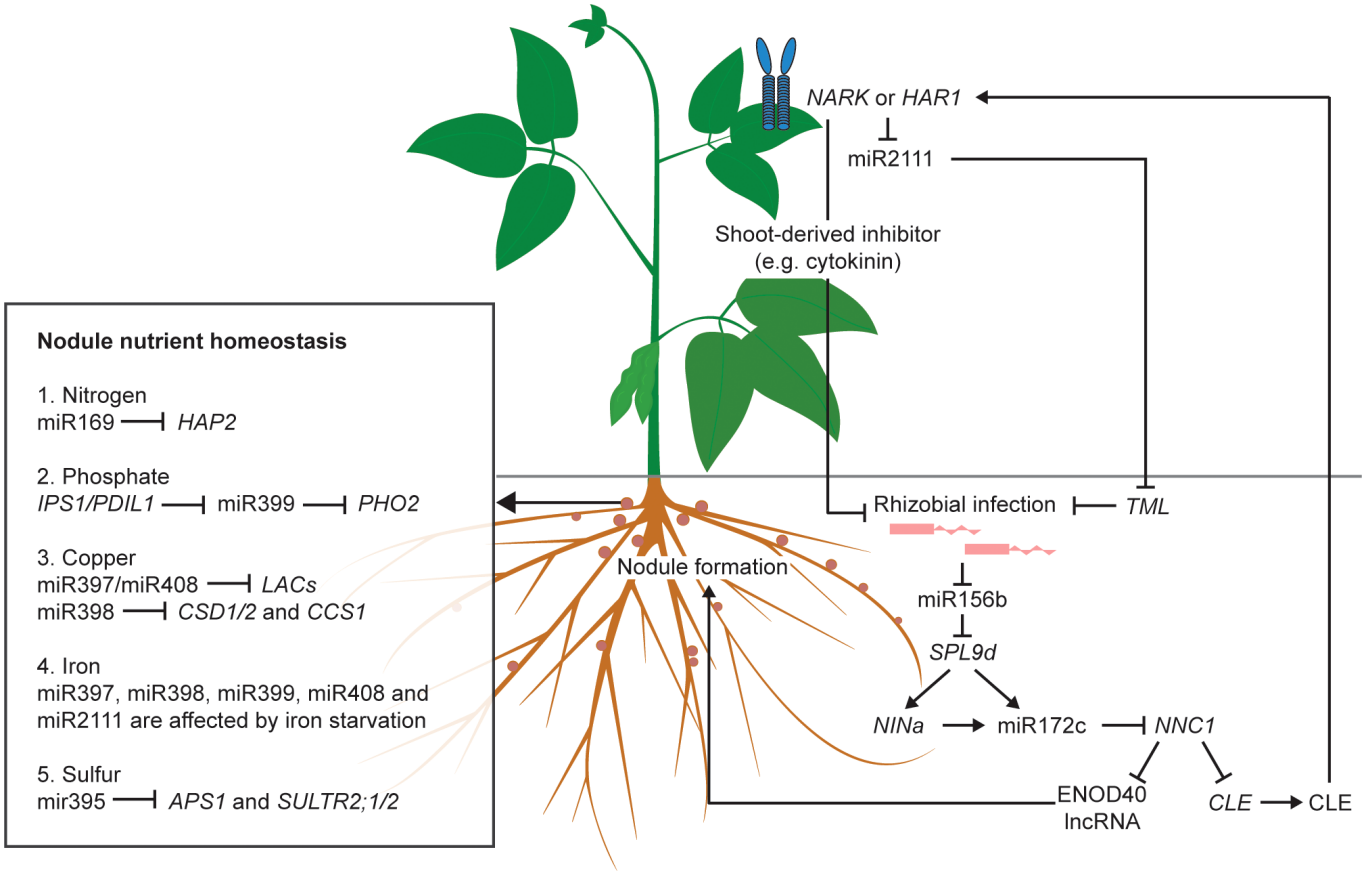
Roles of validated non-coding RNAs (ncRNAs) in AON pathway and nodule nutrient homeostasis of legumes. Without rhizobial infection, miR2111 is transported from shoot to root to inhibit the symbiosis suppressor *TML*, thus maintaining the legume host at an infection-susceptible default status. Rhizobial infection reduces the expression of miR156b to release its inhibition on the downstream symbiosis activators, such as miR172c and *NINa*. The activation of the long non-coding RNA (lncRNA) of *ENOD40* directly induces nodule formation. The induction of *CLE* produces the CLE peptides which is transported from root to shoot and perceived by the NARK/HAR1 receptor to suppress nodule formation, either by activating the shoot-derived inhibitors, such as cytokinin, or by repressing miR2111, thereby allowing *TML* to inhibit rhizobial infection. In the nodule, miRNAs and lncRNAs regulate nutrient homeostasis, such as those of nitrogen, phosphate, copper, iron and sulfur. Arrows indicate activation. Blunt ended arrows indicate inhibition.

On the other hand, the inhibition of *NNC1* by miR172c also upregulates the expression of *Rhizobia-Induced CLE*s (*RIC*s), resulting in the activation of AON (Wang et al., 2019). Following processing, CLEs are transported to the shoot in the xylem, and perceived by a homodimeric or heterodimeric receptor complex in the parenchymal cells of the leaf vasculature, such as NARK (Nodule autoregulation receptor kinase) (reviewed by Ferguson et al., 2019). The perception of RICs activates a shoot-derived inhibitor that travels through the phloem to the root to suppress nodulation (Mortier et al., 2010; Sasaki et al., 2014). The precise identity of this shoot-derived inhibitor (SDI) is still unknown. So far, miR2111 is considered one possible candidate for SDI (Sasaki et al., 2014). When there is no rhizobial infection, miR2111 is maintained at a high level and is translocated from shoot to root to inhibit *Too Much Love* (*TML*), a negative regulator of nodulation, thus maintaining the legume at a default susceptible status (Tsikou et al., 2018). The CLE perception in the leaf suppresses miR2111 expression, leading to the accumulation of *TML* in roots and the subsequent inhibition of nodule organogenesis (Zhang et al., 2021). Taken together, both the upstream and downstream portions of the AON pathway are under the control of ncRNAs. A schematic of the regulation of AON by ncRNAs is shown in **Figure 1**.

## Roles of ncRNAs in nutrient homeostasis to maintain efficient nitrogen fixation

Nitrogen fixation is critical for legume growth. In soybean, nitrogen supplied by biological nitrogen fixation (BNF) is about 52% of the total nitrogen uptake (Salvagiotti et al., 2008). The legume host provides carbon and other nutrients, and the bacteroid supplies the legume with ammonium and amino acids in exchange (Udvardi and Day, 1997). Sufficient nutrient supply from the legume host to the bacteroid is critical for effective nitrogen fixation (Snowball et al., 1980). Many mineral nutrients, such as phosphate, nitrogen, sulfur, copper and iron, are required for high-efficiency nitrogen fixation, (Raei et al., 2013; González-Guerrero et al., 2014; Sulieman and Tran, 2015). NcRNAs, especially miRNAs, are key regulators of nutrient homeostasis in plants, by controlling various nutrient transporters (reviewed in Paul et al., 2015). The nutrient deficiency-responsive ncRNAs and their regulation on nutrient homeostasis are listed in **Figure 1** and **Table 2**.

**Table 2:** NcRNAs involved in nutrient starvation responses.

Type of ncRNAs	Name	Target	Nitrogen	Phosphate	Copper	Iron	Sulfur
miRNA	miR156	*SPL* Transcription Factor	Induced	Induced			Induced
	miR160	*ARF10/16/17*	Induced	Induced			Induced
	miR164	*NAC* Transcription Factor	Induced	Induced			Induced
	miR167	*ARF8a/b*	Suppressed	Induced			Induced
	miR169	*HAP2*	Induced				Induced
	miR171	*GRAS* Transcription Factor	Induced				
	miR172	*AP2*	Suppressed	Induced			
	miR319	*TCP4*	Induced	Suppressed			
	miR395	*APS1* and *SULTR2;1/2*	Suppressed	Suppressed			Induced
	miR397	*LAC*			Induced	Suppressed	
	miR398	*CSD1/2*			Induced	Suppressed	
	miR399	*PHO2*	Suppressed	Induced	Induced	Suppressed	Induced
	miR408	*LAC*		Suppressed	Induced	Suppressed	
	miR826	*AOP2*	Induced				
	miR827	*NLA* and *PHT5*	Suppressed	Induced			
	miR846	Jacalin lectin family genes	Induced				
	miR2111	*TML*		Induced	Induced	Suppressed	
lncRNA	*IPS1*	miR399		Induced			
	*PDIL1*	miR399		Induced			
	*PDIL2/3*	Phosphate transporters		Induced			
circRNA	Chr01:7660079|7660536	miR-RH83					

### Nitrogen

Nitrate negatively regulates all phases of nodulation, including rhizobial infection, nodule formation and development, and nitrogen fixation (Stephens and Neyra, 1983; Kanayama et al., 1990). Until now, many nitrogen starvation-responsive (NSR) miRNAs in plants were identified. For example, miR156, miR160, miR169, miR171, miR319, miR826 and miR846 were induced under low-nitrogen conditions, while miR167, miR172, miR395, miR399 and miR827 were suppressed (Liang et al., 2012; Paul et al., 2015). These NSR miRNAs were also differentially expressed in soybean upon nodulation. For instance, miR156, miR160, miR167, miR171, miR172, miR319, miR395 and miR399 were differentially expressed among uninfected roots, infected roots and nodules in soybean (Fan et al., 2021), suggesting that they also played roles in regulating the nitrogen status in nodules. In roots, miR169-*NF-YA* modules are important for the primary root growth and lateral root initiation (Sorin et al., 2014). In soybean, miR169c was induced by high nitrogen levels to target the transcription factor, *GmNFYA-C* (Xu et al., 2021). Overexpression of miR169c significantly reduced nodule number, whereas knockout miR169c promoted nodule formation (Xu et al., 2021). *GmNFYA-C* was proved to directly induce the expression of *ENOD40* and attenuate the suppression of nodulation by nitrogen (Xu et al., 2021). In Medicago, miR169 was mainly expressed in nodules and targets *MtNF-YA1*, a central transcription regulator of symbiotic nodule development (Combier et al., 2006). *MtNF-YA1* could control meristem persistence and potentially be involved in the symbiotic bacterial release (Combier et al., 2006). Overexpression of miR169 blocked the nodule development by targeting *MtNF-YA1* (Combier et al., 2006). MiR169 exhibited a critical spatial and temporal restriction of *MtNF-YA1* to the nodule meristematic zone to ensure the correct tissue identity and the transition from meristematic to differentiated cells (Combier et al., 2006). In *Lotus japonicus*, *NF-YA1* is presumably under the regulation of *NIN*. *NF-YA1* regulates the expression of the *Short Internode/Stylish* (*STY*) family transcript factors. In turn, *STY* regulates *YUCCA1* and *YUCCA11*, two transcription factors that corresponding to the auxin biosynthesis (Shrestha et al., 2021). This regulatory circuit contributed to the auxin signaling important for the nodule differentiation (Shrestha et al., 2021). In *Lotus japonicas*, miR171c took part in bacterial infection in nodules by targeting the transcript of a transcription factor, *NSP2* (De Luis et al., 2012). Accumulation of miR171c inhibited the formation of infection threads and symbiotically active nodules, whereas the miR171c-resistant *NSP2* could restore the changes (De Luis et al., 2012). In soybean, miR171o and miR171g functioned as negative regulators for nodulation inhibiting the expression of *NIN*, *ENOD40* and Ethylene Response Factor Required for Nodulation (*ERN*) by targeting *SCL-6* and *NSP2* (Montgomery et al., 2008).

### Phosphate

There is a high demand for phosphate in the legume nodule during nitrogen fixation (Gunawardena et al., 1992). Phosphate is commonly incorporated into organic molecules, such as ATP, nucleic acids and enzymes. Deficiency in phosphate leads to shortage in ATP supply, which reduces the efficiency of energy-intensive nitrogen fixation in nodules (Vance et al., 2000; Thuynsma et al., 2014). Under phosphate-deficient conditions, 25 miRNAs are induced and 11 miRNAs are repressed in soybean (Xu et al., 2013). Among the 25 miRNAs, three miRNAs (miR399, miR2111 and miR159e-3p) are also important regulators in nodulation (Xu et al., 2013; Tsikou et al., 2018; Fan et al., 2021). For example, miR2111 is induced under phosphate stress and targets *TML* to regulate nodule formation (Xu et al., 2013; Tsikou et al., 2018). MiR399 was highly induced in soybean roots and nodules after inoculation with *Sinorhizobium fredii*, and could enhance the symbiotic soybean phosphate uptake by suppressing the *phosphate over accumulator 2* (*PHO2*) (Fan et al., 2021). PHO2 is a negative regulator of phosphate transporters responsible for phosphate uptake (Dong et al., 1998; Branscheid et al., 2010; Du et al., 2018). Thus, the upregulation of miR399 upon rhizobium inoculation could relax the inhibition on phosphate uptake. This improvement in phosphate uptake by miR399 can translate into higher ureide production and better overall plant growth (Fan et al., 2021).

Apart from miRNAs, lncRNAs also play a part. An lncRNA, *Induced by Phosphate Starvation 1* (*IPS1*), was also induced by phosphate starvation and functioned as an miR399 sponge (Franco-Zorrilla et al., 2007). *IPS1* harbors a motif with a reverse-complementary sequence to miR399, but with a mismatched loop at the expected miR399 cutting site, which makes *IPS1* not cleavable and instead serves to sequester miR399 (Franco-Zorrilla et al., 2007). Therefore, *IPS1* competitively binds to miR399 to dampen the control by miR399 on *PHO2* (Franco-Zorrilla et al., 2007; Xu et al., 2013). In soybean, four *IPS1* members were found by blast search in the genome and showed a potential role in the regulation of miR399 (Xu et al., 2013).

In *Medicago truncatula*, 10,785 lncRNAs were identified in roots and leaves, and some of them were responsible for the phosphate starvation response (Wang T. et al., 2017). An lncRNA, *Phosphate Deficiency-Induced lncRNA* (*PDIL*) 1 also targets miR399, performing the same function as *IPS1* (Wang T. et al., 2017). Another two lncRNAs, *PDIL2* and *PDIL3*, directly target inorganic phosphate (Pi) transporters at the transcriptional level to maintain phosphate homeostasis (Wang T. et al., 2017).

### Copper

Copper (Cu) is an indispensable element for nitrogen fixation. Sufficient copper supply increases nitrogen fixation efficiency per nodule and elevates the nitrogen content in plants. Copper is a cofactor of metabolic enzymes and protein complexes in the electron transport chain in *Bradyrhizobium japonicum* (Preisig et al., 1996). Copper is also a substrate of several superoxide dismutases involved in oxidative stress responses, which could affect the penetration of the rhizobial infection thread into the legume host (Rubio et al., 2007). In Arabidopsis, the expressions of miR397, miR398, and miR408 are responsive to copper starvation. However, the induction of these miRNAs is abolished in the *spl7* mutant (Abdel-Ghany and Pilon, 2008; Pilon et al., 2009; Pilon, 2017). These three miRNAs could reduce the biosynthesis of non-essential copper-containing enzymes to conserve copper resources among various groups of copper-containing proteins in order to maintain copper homeostasis (Gifford et al., 2008). The expression of miR397 is increased in the nitrogen-fixing nodules but not in the inactive nodules, and miR397 is involved in the nitrogen fixation-related copper homeostasis (De Luis et al., 2012). MiR397 targets the transcripts of copper-containing laccases (LACs), such as *LAC2*, *LAC4* and *LAC17* (Abdel-Ghany and Pilon, 2008). Furthermore, *LAC3*, *LAC12* and *LAC13* are under the control of miR408 (Gifford et al., 2008), which has a higher expression level in soybean nodules than in roots (Fan et al., 2021). MiR398 is induced in common bean nodules under copper deficiency, and is responsible for copper ion availability by reducing the production of non-essential copper-containing enzymes (Naya et al., 2014). It targets *Cu/Zn Superoxide Dismutase 1* and *2* (*CSD1* and *CSD2*) and *Cu chaperones for superoxide dismutase SOD1* (*CCS1*), and is also linked to plastocyanin (Sunkar et al., 2006; Beauclair et al., 2010; Juszczak and Baier, 2012; Naya et al., 2014). CSD1, CSD2, CCS1 and plastocyanin are the most abundant copper-binding proteins in chloroplasts (Abdel-Ghany, 2009; Juszczak and Baier, 2012).

### Iron

Iron is critical for both the legume host and the rhizobium, and has a positive effect on nodulation. Iron deficiency leads to a reduction in nodule numbers in *Lupinus angustifolius* L, and affects nodule development in soybean and common bean (*Phaseolus vulgaris* L.) (Tang et al., 1990; Soerensen et al., 2008; Slatni et al., 2011). The iron concentration in the nodule is positively related to the rate of nitrogen fixation in common bean (Slatni et al., 2008). In Arabidopsis, Iron deficiency-responsive *cis*-Element 1 and 2 (IDE1/2) were identified in 24 miRNA gene promoter regions (Kong and Yang, 2010). Among the 24 miRNAs genes, 17 of them exhibited were differentially expressed under iron starvation (Kong and Yang, 2010). MiR397, miR398, miR399, miR408 and miR2111 were down-regulated by iron deficiency while up-regulated by copper deficiency (Waters et al., 2012). A possible explanation for this is that, under low copper availability, the function of Cu/Zn superoxide dismutases is replaced by iron superoxide dismutase (Waters et al., 2012; Paul et al., 2015). MiR159, miR164, miR172, miR173 and miR394 were also identified to be iron-responsive miRNAs, based on the small RNA sequencing result (Kong and Yang, 2010; Waters et al., 2012). As stated above, most of these iron-responsive miRNAs are important nodulation regulators.

### Sulfur

Sulfur (S) is a critical nutrient for plant growth as a component of amino acids, cofactors, enzymes and secondary metabolites (reviewed by Davidian and Kopriva, 2010; Becana et al., 2018). Sulfur deficiency leads to a reduction in nitrogen fixation, lower nitrogenase activity and the slowing down of nodule metabolism (Anderson and Spencer, 1950; Scherer et al., 2006). Not surprisingly, sulfur starvation inhibits nodulation, such as reducing nodule numbers and nodule mass per unit root length (Varin et al., 2010). Sulfur starvation affects the expression of miR156, miR160, miR167, miR164, miR168, miR394 and miR395 in *Brassica napus* (Huang et al., 2010). MiR395 is the main miRNA responsible for sulfur uptake and translocation (Liang et al., 2010; Yang et al., 2022). It targets and inhibits the expression of the ATP sulfurylase gene, *APS1*, and the sulfate transporter genes, *SULTR2;1* and *SULTR2;2* (Liang et al., 2010). APS1 is responsible for sulfur assimilation, and SULTR2;1 and SULTR2;2 function in sulfur translocation (Liang et al., 2010; Matthewman et al., 2012; Yang et al., 2022). In Arabidopsis, the overexpression of miR395 resulted in the over-accumulation of sulfur in shoots, while a reduced expression of miR395 caused a decline in sulfate concentration (Ai et al., 2016). A high level of miR395 in soybean nodules may ensure higher sulfur accumulation in nodules (Fan et al., 2021). Hydrogen sulfide (H_2_S) in nodules improves the soybean-rhizobium symbiosis and enhances nitrogen fixation (Zou et al., 2019). In *Phaseolus vulgaris*, a circRNA (Chr01:7660079|7660536) potentially functions as a sponge to sequester miR-RH83, to prevent the interaction during symbiosis between miR-RH83 and its target gene, *Phvul.008G170800*, which encodes a sulfur transporter (Wu et al., 2020). Therefore, circRNA (Chr01:7660079|7660536) may play a role in sulfur accumulation in the nodule.

## Future perspectives

Non-coding-RNAs, especially miRNAs, are involved in many aspects of nodulation and nitrogen fixation in legumes. MiR160, miR167, miR390 and miR393 are critical regulators for auxin signaling during the legume nodulation process, responsive to nutrient starvations and required for the enhanced tolerance of nodules to many biotic and abiotic stresses. MiR156 works as an upstream master regulator of legume nodulation by affecting multiple hormone signaling pathways, maintaining nutrient homeostasis and regulating the AON pathway. For the AON pathway, miR172c showed an indispensable role in controlling the expression of both CLE and *ENOD40*. As the lack of functional studies of lncRNAs and circRNAs in legumes, only a few lncRNAs were well studied, like *IPS1* and *PDIL* are controlling the Pi homeostasis. These ncRNAs act as key regulators for the nodulation process and are promising targets for legume crop improvement.

The regulation of nodulation and nitrogen fixation is complicated. Elimination of important regulator may lead to detrimental effects. However, a small change in the expression or specificity of some miRNAs, such as miR172c, may potentially help loosening the AON to improve nodule formation or improving nitrogen fixation. CRISPR/Cas9 system is a commonly used genome editing tool in biology research and is also applied in legume crops, such as soybean, cowpea and chickpea (Chang et al., 2016; Gao, 2018). Apart from complete knockout of target ncRNA, CRISPR/Cas9 system could also be adopted to knockdown ncRNA. Single-guide RNAs (sgRNAs) were designed to target biogenesis processing sites of selected miRNA, the cutting sites of Dicer-like RNase III endonucleases (DCLs) (Chang et al., 2016). The CRISPR/Cas9 construct with this kind of sgRNAs could reduce the expressions of selected miRNA up to 96% (Chang et al., 2016). On the other hand, CRISPR/Cas base editors can be used to alter the base of the miRNAs or their targets in order to fine-tuning the miRNA-target specificity. Furthermore, CRISPR activation system (Pan et al., 2021) may also be adopted to activate the expression of miRNAs such as miR395 and miR399 to improve the nutritional status of the nodules. Apart from CRISPR/Cas9, short tandem target mimic technology (STTM) is also a high-efficient technology to block small RNA functions in plants (Tang et al., 2012). In rice, a large collection of STTM lines silencing 35 miRNA families was established (Zhang et al., 2017). Blocking miR530 was proved to improve the rice grain yield (Sun et al., 2020). Based on the analysis result of the genetic variation, the miR530 locus was regarded as a potentially artificial selection site in rice breeding (Sun et al., 2020).

With the advance in sequencing technologies, the discovery of different classes of ncRNAs is no longer challenging. Nevertheless, investigation of the biological functions of ncRNAs is difficult, especially for lncRNAs. Unlike protein-coding genes, the sequences of lncRNAs show little homology across species. Therefore, predicting the functions of lncRNAs by homology is not feasible. Since there is no functional domain/motif on ncRNAs, functional annotation of ncRNA is also not practical. As the concept of frameshift mutation does not apply to ncRNAs, small INDELs (insertions/deletions) created by traditional genome editing methods are not sufficient to knockout the ncRNAs unless they hit upon some critical nucleotides. Large-fragment deletions or substitutions by more advanced genome editing protocols may be able to solve the problem, but they still require some optimization. Therefore, although hundreds and thousands of ncRNAs have been identified, knowledge of their functions is still limited. Since nodules and roots are buried under the soil, mutant phenotypes are less observable than above-ground phenotypes. Hence, studying the functions of ncRNAs in nodulation and nitrogen fixation requires extra effort.

Plants can take up exogenous miRNAs and siRNAs to activate the RNA interference (RNAi) machinery (Betti et al., 2021). For example, the exogenous application of miR399 and miR156 could trigger RNAi by a mechanism requiring both AGO1 and RDR6 to cause the cleavage of their target genes, *PHO2* and *SPL* (Betti et al., 2021). MiRNAs produced by plants can also be secreted into the environment to affect the gene expressions of the nearby plants, acting as signaling molecules that enable communications between plants (Betti et al., 2021). Actually, several studies have demonstrated that exogenous double-stranded RNAs (dsRNAs), short interfering RNAs (siRNAs), or hairpin RNAs (hpRNAs) could trigger the plant RNAi machinery to protect themselves against plant pathogenic viruses (Gan et al., 2010; Konakalla et al., 2016; Das and Sherif, 2020; Vadlamudi et al., 2020). While the application of GMO is hotly debated, altering plant phenotypes through exogenous applications of double-stranded miRNAs (ds-miRNAs), siRNAs, dsRNAs and hpRNAs may be more acceptable by society and eco-friendlier and more sustainable. Another possibility was the use of rich genetic resources. Nowadays, the large volume of sequencing data allows the discovery of genetic variations of ncRNA in different accessions within the same species. The natural gain or loss of ncRNA loci or natural variation of ncRNA sequence may play role in the regulation of the nodule function. Introduction or elimination of these loci through breeding may also be a viable strategy to improve the nitrogen fixation capability of elite cultivars.

Recently, the miRNA, Pmic_miR-8, was found to be transported from a fungal symbiont, *Pisolithus microcarpus*, to the roots of its symbiotic host, *Eucalyptus grandis* (Wong-Bajracharya et al., 2022). Pmic_miR-8 may inhibit the host transcripts encoding an NB-ARC domain-containing disease resistance protein, to stabilize the symbiotic interaction (Wong-Bajracharya et al., 2022). In rhizobium, three transfer RNA (tRNA)-derived small RNA fragments (tRFs) target the host legume genes associated with the nodulation process by triggering the host RNAi machinery (Ren et al., 2019). These three tRFs, tRFs-dubbed Bj-tRF001, Bj-tRF002, and Bj-tRF003 are the predominant products come from three tRNAs: Val-1-tRNA (CAC), Gly-1-tRNA (UCC), and Gln-1-tRNA (CUG), respectively (Ren et al., 2019). Bj-tRF001, Bj-tRF002, and Bj-tRF003 were predicted to respectively target *GmRHD3a/GmRHD3b*, *GmHAM4a/GmHAM4b* and *GmLRX5*, and hijack the soybean GmAGO1b to cause the tRF-guided cleavage of targets genes (Ren et al., 2019). *GmRHD3a/GmRHD3b*, *GmHAM4a/GmHAM4b* and *GmLRX5* are orthologs of the *Arabidopsis ROOT HAIR DIRECTIVE 3* (*RDH3*), *HAIRY MERISTEM 4* (*HAM4*), and *LEUCINE-RICH REPEAT EXTENSION-LIKE 5* (*LRX*5), which are required for root hair and plant development in *Arabidopsis* (Ren et al., 2019). Gain and loss of function analyses proved that *GmRHD3a/GmRHD3b*, *GmHAM4a/GmHAM4b* and *GmLRX5* negatively regulate the deformed and curled root hairs upon rhizobium infection and the nodule numbers (Ren et al., 2019). Therefore, rhizobial tRFs could promote rhizobial infection (Ren et al., 2019). These studies hinted that legume-rhizobium symbiosis may not be only controlled by the ncRNAs produced by the legume host, but also by the rhizobium-produced small ncRNAs. A more complicated cross-kingdom sRNA gene-silencing network could be involved in the symbiosis between legume and rhizobium, and this requires further exploration.

The picture of the functions of ncRNAs on legume-rhizobium interactions is far from complete. Further delineation of the functions of ncRNAs in the legume-rhizobium symbiosis is important for legume crop breeding and improvement. Artificial manipulations and exogenous applications of ncRNAs could be effective ways to make nodulation and nitrogen fixation more efficient, which in turn can provide a good solution for sustainable agriculture.

## Author contributions

KF, M-WL, and H-ML conceptualized the manuscript. KF, C-CS wrote the first draft. KF, M-WL, and H-ML reviewed and edited the manuscript. All authors contributed to the article and approved the submitted version.

## Funding

The work was supported by Hong Kong Research Grants Council Area of Excellence Scheme [AoE/M-403/16] and Lo Kwee-Seong Biomedical Research Fund for H-ML.

## Acknowledgments

Ms. Jee Yan Chu copy-edited the manuscript. Any opinions, findings, conclusions, or recommendations expressed in this publication do not reflect the views of the Government of the Hong Kong Special Administrative Region or the Innovation and Technology Commission.

## Conflict of interest

The authors declare that the research was conducted in the absence of any commercial or financial relationships that could be construed as a potential conflict of interest.

## Publisher’s note

All claims expressed in this article are solely those of the authors and do not necessarily represent those of their affiliated organizations, or those of the publisher, the editors and the reviewers. Any product that may be evaluated in this article, or claim that may be made by its manufacturer, is not guaranteed or endorsed by the publisher.
